# Prevalence and zoonotic risk factors of *Mycobacterium bovis* tuberculosis in cattle at the cattle-wildlife-human interface in South and East Cameroon

**DOI:** 10.14202/vetworld.2024.8-16

**Published:** 2024-01-04

**Authors:** Armelle Prudence Kouengoua Kouengoua, Yves Ledoux Tsissa, Nestor Denakpo Noudeke, Roland Nankam Chimi, Arouna Njayou, Abdou Karim Issaka Youssao, Mahamadou Dahouda, Cyrille Boko, Victorien Dougnon, Julius Awah-Ndukum, Farougou Souaibou

**Affiliations:** 1 Department of Veterinary Public Health, Faculty of Veterinary Medicine and Agriculture, Universite des Montagnes Bangangte, Cameroun; 2 Research Unit on Transmissible Diseases-Ecole Poly Technique, University of Abomey-Calavi, Benin; 3 Department of Biomedical Science, Faculty of Science, University of Ngaoundéré, Cameroon; 4 Research Unit in Applied Microbiology and Pharmacology of Natural Substances, University of Abomey-Calavi, Benin; 5 Department of Animal Production Technology , College of Technology, University of Bamenda, Cameroon

**Keywords:** bovine tuberculosis prevalence, East and South Cameroon, livestock-wildlife-human interface, zoonotic risk factors

## Abstract

**Background and Aim:**

Bovine tuberculosis (bTB) is a contagious and notifiable disease, which is prevalent in cattle populations of many countries and in several wildlife species worldwide. However, the role of wildlife in the transmission and/or maintenance of bTB at the human-wild animal-animal interface and the epidemiology of zoonotic disease are poorly understood in Cameroon, where many wildlife species exist. This study aimed to estimate the prevalence and zoonotic risk factors of bTB at the cattle-wildlife-human interface in the South and East regions of Cameroon.

**Materials and Methods:**

We conducted a descriptive cross-sectional study from May to October 2022 in the southern region (Vallée du Ntem and Dja et Lobo) and eastern region (Haut Nyong and Lom et Djérem) of Cameroon to determine risk factors for bTB in Zebu Bororo, Goudali, Ndama, and Simmental cattle breeds. A comparative intradermal tuberculin testing (CIDT) was performed on 160 cattle randomly selected from herds using the threshold recommended by the World Organization for Animal Health. An interviewee-administered questionnaire was used to gather epidemiological data on sociodemographics, interaction between cattle and wildlife, and awareness of zoonotic tuberculosis (TB) from 90 cattle professionals. The prevalence of bTB at the herd level and associated risk factors were estimated using multiple logistic regression models.

**Results:**

Based on the comparative intradermal tuberculin test (CIDT), the estimated prevalence of bTB in 160 cattle (Zebu Bororo, Goudali, Ndama, and Simmental) in South and East Cameroon was 6.8% (4.35%–9.41%) and 1.8% (0%–3.6%) for threshold values 3 mm and 4 mm, respectively. The prevalence obtained by simple intradermal tuberculin test (IDT) was 0.6% (0%–1.2%) for a threshold value 4 mm. Univariate analysis revealed three risk factors associated with bTB with significant odds ratios (OR; p = 0.05): herd size (OR = 4.88; 95% confidence interval [CI]: 1.24–32.56); cattle aged>10 years (OR = 0.17; 95% CI: 0.05–0.53); and victims of bTB organ seizure (OR = 0.015; 95% CI: 0.002–0.067). Multivariate analysis showed that being a cattle herder and contact between wildlife and livestock due to forage was significantly associated with bTB exposure (adjusted OR = 0.02; p = 0.001).

**Conclusion:**

Bovine TB is prevalent in cattle of the South and East Cameroon. Comparative IDT of cattle reared in the epidemiological and environmental context of the study areas yielded better results at a threshold of 3 mm than at a threshold of 4 mm recommended by the World Health Organization. Factors associated with exposure to/appearance of bTB were high herd size, cattle aged >10 years old, seizures of tuberculous organs, shepherding as a profession, and contact between cattle and wildlife can be due to lack of forage.

## Introduction

Bovine tuberculosis (bTB) is a chronic bacterial disease in animals caused by *Mycobacterium tuberculosis* complex members, mainly *Mycobacterium bovis*. bTB is a major zoonotic disease, and cattle are the main source of infection for humans. It also affects other domesticated animals, such as sheep, goats, equines, pigs, dogs, and cats, as well as wild boar, deer, and antelope [1]. Infection in humans is most often caused by direct contact with infected animals as well as the consumption and handling of animal products and by-products [1]. Bovine TB is present in all parts of the world with varying frequency. For example, the prevalence in Northwest Ethiopia is 9.1% [2]. In developed countries, the cost of bTB is mainly related to losses in livestock production, including increased mortality and lower milk and meat production. Estimates of such losses have been made for countries with a large livestock population, such as Ethiopia [3]. A comparative intradermal tuberculin test (CIDT) reported the prevalence of *M. bovis* TB among dairy cows in Egypt at 68.75% (95% confidence interval [CI]: 46–91.4) and 1.67% (95%, CI: 1.3–2.1) in 2020 [4]. Poor herd management, history of bTB infection, and introduction of new animals into the herd were identified risk factors. The prevalence of bTB in dairy cows in Eastern Ethiopia was 20.3% (n = 64) according to CIDT at a threshold >4 mm. Knowledge of *M. bovis* TB was 33%; 23% of participants had knowledge of the zoonotic nature of animal TB; 50% preferred to consume raw milk; and 7% had direct contact with dairy cows [5]. Bovine TB is endemic in Cameroon, but poorly evaluated [6].

bTB is a legally contagious and notifiable disease [7]. The role of wildlife in the transmission and/or maintenance of bTB in certain domestic outbreaks is currently a subject of debate. In Africa, *M. bovis*-induced TB is prevalent in cattle populations of many countries and in several wildlife species, including the African buffalo, lion, baboon, kudu, warthog, and others [1, 8]. The epidemiology of zoonotic diseases is poorly understood, and transmission occurs at the human-wild animal-animal interface, making it difficult to eradicate [9]. Cameroon has many wildlife species. Bovine TB is endemic in cattle in Cameroon and continues to be detected in slaughterhouses during meat inspections [10]. Given the involvement of wildlife and the significant economic and public health importance of bTB [11], it should be viewed with greater vigilance.

Given the limited information available on the occurrence of *M. bovis* TB in wildlife and its circulation at the livestock-wildlife-human interface, a study was conducted to estimate the prevalence and zoonotic risk factors of *M. bovis* TB in cattle in the southern and eastern regions of Cameroon.

## Materials and Methods

### Ethical approval and Informed consent

Risk assessments of the project were performed by the researchers to avoid hazards to all persons and animals involved in the project. Permission for the study was obtained from the required authorities and Local Ethical Committees in the South Region, Cameroon including the Regional Delegation of Livestock, Fisheries and Animal Industries, Regional Delegation of Public Health, Faculty of Veterinary Medicine and Agriculture of Universite des Montagnes-Bangangte, Cameroon. These bodies also provided the research with staff to facilitate data collection during the study. The purpose of the study was explained (with the assistance of local veterinary practitioners, community leaders and trusted intermediaries) to cattle professionals and were used in the study after giving their verbal informed consent.

### Study period and location

**Figure-1:**
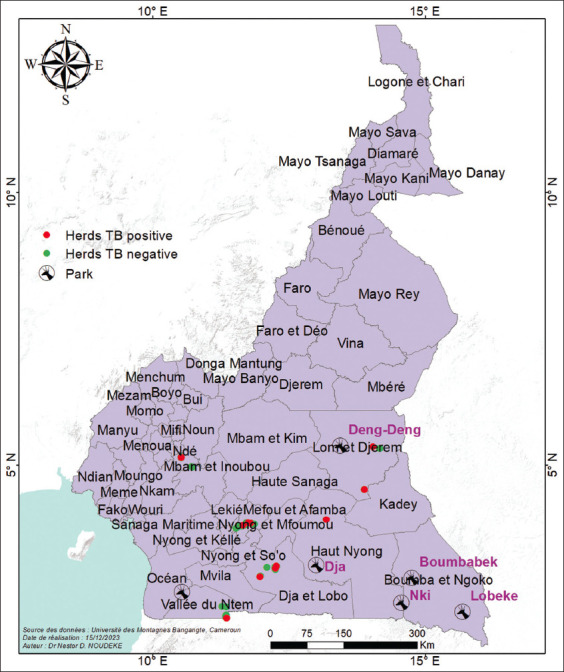
Identification of study animal near/away from protected areas [Source: The map was generated using ArcGis10.8].

This study was conducted from May to October 2022 in the South and East regions of Cameroon. South Cameroon, the capital of which is Ebolowa, lies between 2° and 30° N latitude and between 11° and 45° E longitude. It is bordered to the northwest by the coastal region, to the south by the central region and to the east by the eastern region. The southern part is bordered by three countries: Equatorial Guinea, Gabon and the Democratic Republic of Congo. The southern region has an area of 47,110 km^2^ and a population of 920,715. The eastern region of Bertoua has latitude of 4° N and a longitude 14° E. It is bordered to the south, Adamawa to the north and central regions. The eastern part covers an area of 109,002 km^2^ and has 771,755 inhabitants. The animal study sites are represented in Figure-1.

### Materials

Syringes with refillable reservoirs, shearing scissors, caliper, alcohol, bovine and avian tuberculin, paint, and gloves were used during the intradermal tuberculin test (IDT). A survey form was completed for data collection on sociodemographic and animal-human interactions.

### Study animal

For the comparative intradermal tuberculin skin test (CIDT), cattle above 6 months of age with no clinical signs of disease were included. Study animal-related information, such as sex, age, and body condition score, were collected and recorded at the time of the test for each tested cattle. The sample size was determined using the non-random technique according to the following formula [12]:


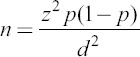


n represents the required sample size; z, the 95% CI (standard deviation value of 1.96); d, the 5% margin of error; and p, the average bTB prevalence of 5.53% [6] in the highlands of Cameroon, from cattle was considered. The minimum farm size was estimated to be 81 farms.

Seventeen farms were sampled: Eight in the south (Ntem valley and Dja et Lobo) and nine in the east (Haut Nyong and Lom et Djérem). In each farm, approximately nine cattle were identified according to the shepherds’ advice.

### Intradermal tuberculin testing

A comparative intradermal tuberculin test (CIDT) was carried out to estimate the prevalence of bTB as described in the World Organization for Animal Health (WOAH) Manual of Diagnostic Tests and Vaccines for Terrestrial Animals [13]. In brief, it is based on the simultaneous use of 20,000 IU/mL bovine purified protein derivative (PPD) AN5 strain PPD-B and 25,000 IU/mL avian-PPD D4 ER strain (PPD-A) from CZ Vaccines (Spain). The initial skin thickness was measured, followed by intradermal injection of bovine and avian-PPD on the right side of the cervical region of the skin at two points approximately 12–15 cm apart. The results were read 72 h later, and the interpretation is based on Table-1 [13].

**Table-1:** Intradermal tuberculin test interpretation grid source [13].

Bovine tuberculin	WOAH manual [13]	Result “reaction”
DB >2 mm	DB–DA >4 mm	Positive
	DB–DA (1–4 mm)	Inconclusive
	DB–DA <1 mm	Negative
DB <2 mm	DB–DA	Negative
SITT	>4 mm	Positive

DB=Variation in skinfold thickness with bovine tuberculin, DA=Variation in skinfold thickness with avian tuberculin, SITT=Simple intradermal tuberculin test, CIDT=Comparative intradermal tuberculin test, WOAH=World organization for animal health

### Determination of risk factors

Interviews were conducted to collect epidemiological data using a pre-tested “closed-ended” questionnaire written both in English and French that could be translated into the participant’s local language on the participant’s request. The interviews were conducted after obtaining the respondent’s consent. Face-to-face interviews lasted for 10–15 min. Data related to herd management, interaction between cattle and wildlife, knowledge on zoonotic TB, and farm’s bTB status were sought. Participants were recruited using the snowball technique. Known cattle professionals may lead to other cattle professionals among their acquaintances.

### Statistical analysis

Microsoft Excel 2010 (Microsoft Office Professional Plus 10, Microsoft Corporation, USA) was used to organize data and IBM SPSS Statistics 20.0 (IBM Corp. Released 2020. IBM SPSS Statistics for Windows, Version 27.0. Armonk, NY: IBM Corp) was used to analyze data. Apparent prevalence (AP) was used as a statistical measure to express the ratio of tuberculous cattle identified by each diagnostic technique to the total number sampled. To estimate the prevalence, the following formula was used:

AP = Number of tuberculous cattle indentified by a method / Total number of cattle sampled

Pearson’s Chi-square was used to evaluate the statistical significance of the associations of different categorical variables with skin test results; and McNemar’s Chi-square was used to assess the association of PPD-A and PPD-B results.

Univariate and multivariate analyses were performed using R 2.10.0 statistical software (R Core Team, Vienna, Austria) to determine the factors that significantly influenced TB status. Odds ratios (ORs) and p-values were calculated. Subsequently, we selected the most suitable model for determining the risk factors. We first run a model with the specified variables and check if the model can be improved by deleting some of these variables. We deleted the variable whose deletion would improve the model the most, that is, we then repeated the same procedure to determine if deleting a second variable could still improve the model, and so on. When the model could no longer be improved by deleting a variable, we terminated the experiment. We defined criteria for determining the quality of a model. The Akaike information criterion (AIC) is one of the most widely used criteria. The lower the AIC value, the better the model. Therefore, after the univariate analysis, all explanatory variables were taken back for a top-down step-by-step analysis to determine the final model. As soon as the main effects have been identified, all possible interactions with the final model have also been examined. We quantified the strength of the association between all variables by evaluating the ORs and corresponding 95% CIs. We quantified the contribution of each variable in the final model. Multi-collinearity was also tested using the variance inflation factor (VIF) of the explanatory variables to avoid model convergence. Multivariate analysis aimed to highlight the factor(s) still present in the logistic regression model despite their combination and presented adjusted ORs (AORs) and p-values.

## Results

Intradermal tuberculin testing revealed one CIDT-positive cattle, three simple intradermal tuberculin test (SITT)-positive cattle at the WOAH recommended threshold of 4 mm, and 11 CIDT-positive cattle at the WOAH recommended threshold of 3 mm [6].

### Overall prevalence of bTB on SITT and CIDT in cattle in the South and East

Table-2 shows the AP of bTB according to the IDT positivity thresholds. Simple intradermal tuberculin test conducted at a threshold of 4 mm identified 3 positive reactions out of 160, giving an AP of 1.8% (95% CI: 0%–3.6%). CIDT performed at a threshold of 3 mm found 11 positive reactions with a prevalence of 6.8% (95% CI: 4.35%–9.41%). Three positive reactions were identified for CIDT performed at a threshold of >4 mm, with a prevalence of 0.6% (95% CI: 0%–1.2%).

**Table-2:** Distribution of apparent bTB prevalence by SITT and CIDT.

IDT	Total (n)	Positive	AP % (95% CI)
SITT			
≥4 mm	160	3	1.8 (0%–3.6%)
CIDT			
≥3 mm	160	11	6.8 (4.35%–9.41%)
≥4 mm	160	1	0.6 (0%–1.2%)

IDT=Intradermal tuberculin test, SITT=Simple intradermal tuberculin test, AP=Apparent prevalence, 95% CI=95% confidence interval, *P<*0.05, bTB=bovine tuberculosis

### Prevalence of bTB on SITT and CIDT according to sociodemographic parameters in cattle

Table-3 shows that body condition score significantly influenced the prevalence of bTB at CIDT threshold 3 mm. Other sociodemographic variables were not significant.

**Table-3:** Distribution of apparent bTB prevalence on sociodemographic parameters.

Variables	Herd size	AP (%) (95% CI)			Comparison of AP for CIDT and SITT Chi-square (p-value)		
	
		CIDT ≥3	CIDT ≥4	SITT ≥4	CIDT ≥3	CIDT ≥4	SITT ≥4
Sex							
Male	66	7.57 (3.55–11.12)	1.51 (0.54–2.05)	4.45 (3.5–7.95)	0.0009 (0.976)	-	-
Female	94	7.44 (3.2–11.67)	0	0			
Breed							
Bororo	13	0	0	0	-	-	-
Goudali	120	9.16 (7.21–11.27)	0.83 (0–1.66)	2.5 (1.4–3.9)			
Ndama	22	0	0	0			
Simmental	5	0	0	0			
Age interval							
<3	37	13.5 (10.55–16.05)	0	5.4 (2.3–8.5)	3.986 (0.136)	-	-
(3–6)	80	6.25 (3.6–9.5)	1.25 (0–2.5)	1.25 (0–2.5)			
>6	43	2.32 (0.9–3.74)	0	0			
Body score condition							
Normal	56	19 (7.52–11.3)	17.85 (10.5–27.3)	5.36 (2.5–7.8)	16.230 (<0.0001*)	-	-
Overweight	104	0.96 (0.3–1.6)	0	0			
Region							
South	75	5.33 (4.2–6.8)	1.33 (0.6–2.3)	1.33 (0.6–2.3)	0.524 (0.469)	-	0.225 (0.635)
East	85	8.23 (5.1–10.97)	0	2.35 (1.2–2.15)			

AP=Apparent prevalence, CI=Confidence interval, SITT=Simple intradermal tuberculin test

### Identification of factors associated with bTB

#### Univariate analysis of factors associated with exposure to/appearance of bTB in herds with some proximity or not to reserves, parks

Table-4 presents the ORs and p-values of the various risk factors associated with bTB Farms with cattle numbers between 21 and 160 were 4 times more exposed to bTB (OR = 4.88; p < 0.05) and cattle aged >10 years were most likely to become exposed to bTB (OR = 0.17; p < 0.05). Furthermore, farms that suffered from tuberculous organ seizure at the slaughterhouse were 3 times more exposed to bTB (OR = 0.015; p < 0.05).

**Table-4:** Factors associated with exposure to zoonotic tuberculosis in cattle.

Variables	Modalities	Tuberculosis status, No, n=73 (82%)	Tuberculosis status, Yes, n=16 (18%)	Odds ratio (95% CI)	p-value
Factors associated with profession (n=5)					
Profession/cattle herder	Yes	54 (84)	10 (16)		0.36
	No	19 (76)	6 (24)	1.71 (0.52–5.26)	
Profession/hunter	Yes	1 (100)	0 (0)		0.99
	No	72 (82)	16 (18)		
Profession/butcher	Yes	22 (73)	8 (27)		0.13
	No	51 (86)	8 (14)	0.43 (0.14–1.31)	
Sex	Female	1 (100)	0 (0)		0.99
	Male	72 (82)	16 (18)		
Region	South	24 (83)	5 (17)		0,9
	East	49 (82)	11 (18)	1.07 (0.35–3.74)
Factors linked to cattle breeding (n=4)					
Local	Yes	26 (87)	4 (13)		0.42
	No	47 (80)	12 (20)	1.65 (0.52–6.41)	
Hybrid	Yes	49 (79)	13 (21)		0.3
	No	24 (89)	3 (11)	0.47 (0.10–1.63)	
Meat breed	Yes	67 (81)	16 (19)		0.99
	No	6 (100)	0 (0)	-	
Dairy breed	Yes	5 (100)	0 (0)		0.99
	No	68 (81)	16 (19)	-
Factors linked to breeding system and herd size (n=16)					
Herd size	5–20 heads	30 (94)	2 (6.2)		0.045*
	21–160 heads	43 (75)	14 (25)	4.88 (1.24–32.56)	
Extensive breeding system	Yes	1 (100)	0 (0)		0.99
	No	72 (82)	16 (18)	-	
Semi-intensive breeding system	Yes	70 (81)	16 (19)		0.99
	No	3 (100)	0 (0)	-	
Intensive breeding system	Yes	2 (100)	0 (0)		0.99
	No	71 (82)	16 (18)	-	
Cattle aged 1–3 years	Yes	12 (100)	0 (0)		0.99
	No	61 (79)	16 (21)	-	
Cattle aged 3–10 years	Yes	42 (89)	5 (11)		0.064
	No	31 (74)	11 (26)	2.98 (0.97–10.27)	
Cattle aged >10 years	Yes	20 (65)	11 (35)		0.00329**
	No	53 (91)	5 (8.6)	0.17 (0.05–0.53)	
Emaciated	Yes	58 (78)	16 (22)		0.99
	No	15 (100)	0 (0)	-	
Low weight	Yes	15 (83)	3 (17)		0.87
	No	58 (82)	13 (18)	1.12 (0.31–5.33)	
Normal	Yes	55 (81)	13 (19)		0.61
	No	18 (86)	3 (14)	0.70 (0.15–2.50)	
Overweight	Yes	4 (100)	0 (0)		0.99
	No	69 (81)	16 (19)	-	
Cattle from heritage	Yes	24 (77)	7 (23)		0.41
	No	49 (84)	9 (16)	0.63 (0.21–1.50)	
Cattle from neighborhood	Yes	48 (86)	8 (14)		0.24
	No	25 (76)	8 (24)	1.92 (0.62–5.82)	
Cattle bought in the market	Yes	3 (75)	1 (25)		>0.9
	No	70 (82)	15 (18)	0	
Cattle grazing at which distance from park boundary	80–150 m from park	33 (85)	6 (15)		0.57
	>150–60 m from park	40 (80)	10 (20)	1.40 (0.46–4.41)	
Factors related to tuberculosis signs (n=5)					
Reasons for livestock-wildlife contact is forage	Yes	0 (0)	2 (100)		0.99
	No	73 (84)	14 (16)	-	
Recognizing the signs of tuberculosis	Yes	57 (78)	16 (22)		0.06
	No	16 (100)	0 (0)	0.10 (<0.01–1.08)	
Cough and breathing difficulties	Yes	58 (78)	16 (22)		0.99
	No	15 (100)	0 (0)	-	
Cases of human tuberculosis	Yes	1 (33)	2 (67)		0.5
	No	72 (84)	14 (16)	14,70 (0.00–65.05)	
Caseum on organs	Yes	57 (78)	16 (22)		0.99
	No	16 (100)	0 (0)	-	
Victim of tuberculous organs seizure	Yes	7 (33)	14 (67)		9.3^e.07***^
	No	66 (97)	2 (2.9)	0.02 (<0.01–0.07)	

#### Identification of variables associated with significant effects on the initial model

For the initial model, 30 factors were analyzed. Table-4 shows significant ORs (p < 0.05) for the three factors, indicating their association with a significant effect on the initial model. Herd size, cattle aged >10 years old, and history of tuberculous organ seizure are shown. There is no close proximity (more than 260 m boundary) between cattle farms and parks and reserves.

#### Multivariate analysis of factors associated with exposure to/appearance of bTB in herds with some proximity or not to reserves, parks

Only variables with p < 0.2 were included in the multivariate analysis. To test the model, variables such as profession/shepherd and contact between livestock and wildlife due to food were also considered.

Table-5 shows that farms victim of seizure of tuberculous organs at the slaughterhouse are 50 times more likely to be exposed to bTB ([AOR = 0.02]; 95% CI: [0.00–0.12]), p < 0.001.

**Table-5:** Multivariate analysis of factors associated with bTB exposure in southern and eastern Cameroon.

Variables	Modalities	Adjusted OR	95% CI	p-value
Profession/cattle herder	Yes	—	—	
	No	5.4	0.83–51.4	0.094
Reason of contact between wild and cattle is forage	Yes	—	—	
	No	0		>0.9
Profession/hunter	Yes	—	—	
	No	0	0.00	>0.9
Cattle aged between 3 and 10 years	Yes	—	—	
	No	0		>0.9
Recognizing signs of TB	Yes	—	—	
	No	0	—	>0.9
Cattle herd size	(5.20)	—	—	
	(20.160)	0.81	0.06–11.2	0.9
Cattle aged >10 years	Yes	—	—	
	No	0		>0.9
Victims of tuberculous organs seizure	Yes	—	—	
	No	0.02	0.00–0.12	<0.001

CI=Confidence interval, OR=Odds ratio, bTB=Bovine tuberculosis

Table-6 shows that the reason for livestock-wildlife contact, victim of bTB organ seizure, had a significant effect on the initial model. p-values associated with the odd ratios indicate whether an OR differs significantly from the reference modality. However, it does not indicate whether a variable has a significant effect on the overall model.

**Table-6:** Identification of variables with significant effects on the initial model.

Variable	Df	Deviance	AIC	LRT	Pr
Sex	1	29.073	83.073	0	1
Local breed	1	29.135	83.135	0.062	0.80338
Herd size	1	29.536	83.536	0.463	0.49621
Group Distance from park	1	29.266	83.266	0.1925	0.66087
Age 3–10 years	1	29.073	83.073	0	0.99999
Heritage	1	29.073	83.073	0	0.99997
Loss of weight	0	29.073	85.073	0	
Cattle herder	1	29.264	83.264	0.1908	0.66226
Hybrid breed	1	29.593	83.593	0.5201	0.47078
Extensive breeding	0	29.073	85.073	0	
Age >10 years	1	29.073	83.073	0	0.99997
Neighborhood	1	29.073	83.073	0	0.99997
Caseum	0	29.073	85.073	0	
Hunter	1	29.073	83.073	0	0.99995
Meat breed	1	29.082	83.082	0.0093	0.92331
Semi-intensive breeding	0	29.073	85.073	0	
Low weight	1	29.073	83.073	0	0.99999
Market	1	29.085	83.085	0.0115	0.91458
TB signs	1	29.073	83.073	0	0.99998
Sell	1	29.074	83.074	0.0009	0.97588
Dairy breed	1	29.073	83.073	0	0.99997
Intensive breeding	0	29.073	85.073	0	
Normal weight	1	29.073	83.073	0	0.99999
Organs seizure	1	51.177	105.177	22.1041	0.000002583***
Forage reason	1	33.474	87.474	4.4006	0.03593*
Region	1	29.677	83.677	0.6039	0.43708
Age 1–3 years	1	29.073	83.073	0	1
Overweight	1	29.122	83.122	0.0494	0.82409
Cough	0	29.073	85.073	0	
TB in humans	1	29.654	83.654	0.5811	0.44588

AIC=Akaike information criterion; LRT=Lamda likelihood ratio statistic, Pr=Associated critical probability, TB=Tuberculosis

Table-7 presents the final model selection results. The original model has an AIC of 85.07. After the first stage, the deletion of various variables reduced AIC to 45.83. The deletion of any other variable would increase the AIC, which would lead to the end of the procedure and the identification of the factors most likely associated with bTB.

**Table-7:** Selection of the final model with the factors most significantly associated with bTB.

Variable	Df	Deviance	AIC
Forage reason	1	38.701	46.701
Cattle herder	1	38.946	46.946
Age >10 years	1	41.013	49.013
Tuberculous organs seizure	1	64.425	72.425

AIC=Akaike information criterion

#### Checking multi-collinearity in the final model VIF

The classic approach to measuring collinearity is to examine the VIF. Variance inflation factors estimate the increase in variance of a coefficient due to a linear relationship with other predictors. Therefore, a VIF of 1.8 indicates that the variance of this particular coefficient is 80% greater than the variance that would have been observed if it had not been correlated with other predictors. No multicollinearity exists when all VIFs are equal to one; however, if some VIFs are >1, the predictors are correlated. There has been no consensus on the value at which multi-collinearity should be considered. Some authors claim that they look more closely at variables with a VIF above 2.5. In the present study, all VIFs were close to one, indicating that collinearity was not explored.

Table-8 shows that the occupation of being a shepherd, cattle aged >10 years old, history of tuberculous organ seizure, and wildlife-livestock contact due to food are factors associated with bTB.

**Table-8:** Multi-collinearity in the final model.

Factor	Forage reason	Cattle herder	Age >10 years	Tuberculous organs seizure
VIF	1	1.212942	1.106897	1.172151

VIF=Variance inflation factor

## Discussion

In this study, we determined the prevalence of *M. bovis* TB in cattle at the cattle–wildlife–human interface in East and South Cameroon and identified the associated risk factors for this disease.

Simple intradermal tuberculin test and CIDT were used as diagnostic methods using the WOAH positive decision threshold 4 mm. To evaluate the diagnostic performance of IDT, this method was reinforced by the techniques previously described in studies in Ethiopia [14] and Taiwan [15]. Our CIDT results at a threshold of 3 mm revealed 11 positive reactions out of 160 cattle (Zebu Bororo, Goudali, Simmental, and Ndama), that is, 6.8% (95% CI: 4.35%–9.41%), compared with three positive reactions out of 160 at a threshold of 4 mm, that is, 0.6% (95% CI: 0%–1.2%). These results showed that a threshold of 3 mm for IDT increased the sensitivity of bTB detection when compared with a threshold of 4 mm for WOAH positivity [13]. Our results corroborate those reported by Ndukum *et al*. [6], who obtained 5.53% prevalence (95% CI: 3.59%–7.48%) at the CIDT threshold of 3 mm in Goudali, Zebu Bororo, and their crossbreds in the highlands of Cameroon (Adamaoua and Northwest). Using the performance values obtained in the present study to calculate and compare true prevalence between thresholds ≥3 mm and ≥4 mm for CIDT and SITT, we found that the AP values of reactions at the positivity threshold ≥3 mm were significantly higher than those at positivity threshold ≥4 mm for CIDT. These results further confirm that the application of the positivity threshold ≥3 mm increased the actual prevalence of bTB in the cattle studied. Furthermore, the use of threshold ≥3 mm increased the significance of the tests (CIDT and SITT) and is suitable to the local conditions in Cameroon. Similar results were previously obtained on zebu cattle in Ethiopia [14], demonstrating that the best bTB diagnostic characteristics determined by CIDT were identified at a positivity threshold strictly >2 mm. This would be another reason to review the positivity threshold for IDT as applicable to the context. In our study, we obtained an AP of 6.8% (95% CI: 4.35%–9.41%) with CIDT. This result is lower (11.8%) than that obtained in Bangladesh in dairy cows [16] and that in Eastern Ethiopia in dairy cows (20.3%) by CIDT [5]. These high prevalence rates in both *Bos indicus* cattle (Zebu Bororo, Goudali, Ndama, and Simmental) and dairy cows in Africa demonstrate to the enzootic nature of the disease. Furthermore, we observed a significant difference between the IDT at threshold >3 mm and body condition score, with higher prevalence rates in healthy animals. These results are in agreement with the higher prevalence rates previously reported for female cattle over 4 years [10]. However, some studies have reported no association between body score and prevalence [17–19]. Positive results for IDT clearly indicate that the disease exists in the study areas. Since bTB is a major zoonosis [13], the risk of human infection with *M. bovis* exists, especially as contact between shepherd and cattle is constant and permanent, creating conditions for cross-infection [11]. *M. bovis* TB is the least managed endemic zoonosis worldwide, particularly in developing countries [10, 20]. In countries where bTB is endemic, such as Cameroon [10], and where control programs are not enforced, the risk of human infection with *M. bovis* is high [20]. Our univariate and multivariate analyses revealed that herd size (>21 head) is a risk factor significantly associated with bTB exposure, whereas a herd size > 10 head was previously identified [16]. Given the contagious nature of bTB, as well as its direct mode of transmission, cattle-cattle transmission is very high. Herd size has repeatedly been reported as a risk factor [21]. Cattle aged >10 years were significantly more exposed to bTB, which was similarly reported in a study in Bangladesh, where cattle aged >6 years were considered at risk [16]. Tuberculosis is a chronic disease, and the manifestation of clinical signs may be the result of a depressed immune system, which sometimes occurs at an age when the immune system is weak, or, in the context of our study, when cattle aged >10 years are taken to slaughterhouses. As a consequence, lesions are most commonly observed after slaughter. Another important factor associated with bTB exposure is the seizure of tuberculous organs. As reported previously [4, 22], this may mean that the source of infection has not been eradicated, which represents a permanent source of bTB re-emergence on the farm [14]. Feeding as a point of contact between cattle and wild animals is significantly associated with bTB exposure. In some countries, such as Mozambique [22], wildlife (buffalo) is considered a bTB reservoir and, therefore, a source of infection for cattle. No correlation was found between wildlife reservoirs and bTB transmission and/or maintenance. The respondents and animals evaluated in this study were considerably far (over 100 km) from the protected areas.

## Conclusion

bTB is present in cattle in the southern and eastern regions of Cameroon. Wild animal populations predominate in these regions, and many communities have a high level of wildlife activities. Our study also highlights the need to define an IDT positivity threshold value that is most suitable to the environmental and epidemiological contexts of Cameroon in order to ensure the efficient detection of bTB in cattle. More specifically, we suggest that thresholds of 3 mm should be applied in Cameroon rather than 4 mm, as the former allows a significant gain in IDT.

## Authors’ Contributions

APKK, YLT, JAN, and FS: Conceived, designed and coordinated the study. JAN and FS: Contributed equally and were the principal investigators. APKK, YLT, and JAN: Designed data collections tools. APKK, YLT, JAN, and FS: Supervised the field sample and data collection, and laboratory work as well as data entry. APKK, YLT, NDN, RNC, AN, AKIY, MD, CB, and VD: Contributed reagents, materials, and analysis tools. APKK, JAN, FS, AN, AKIY, MD, CB, VD, NDN, and RNC: Carried out the statistical analysis and interpretation, and participated in preparation of the manuscript. All authors have read, reviewed, and approved the final version of the manuscript.
